# Japanese encephalitis (JE) mimicking acute ischemic stroke: A case report

**DOI:** 10.1097/MD.0000000000023071

**Published:** 2020-11-06

**Authors:** Jiali Zhao, Fudi Chen, Lin Lu, Chunxia Li, Yifeng Du

**Affiliations:** a Department of Neurology, Shandong Provincial Hospital Affiliated to Shandong First Medical University; b Department of Neurology, Shandong Provincial Hospital Affiliated to Shandong University; c Department of Emergency, Shandong Provincial Hospital Affiliated to Shandong First Medical University, Jinan, Shandong, China.

**Keywords:** Japanese encephalitis, Japanese encephalitis virus immunoglobulin M, magnetic resonance, stroke mimic

## Abstract

**Introduction:**

Japanese encephalitis (JE) is one of the most serious viral infectious diseases of the central nervous system in Asia. The clinical manifestations of it might be non-specific. We herein report a case of JE mimicking acute ischemic stroke.

**Patient concerns:**

A 52-year-old man presented with acute onset of left-sided limb weakness for 2 hours and a 5-year history of hypertension but with no fever or cold before the onset. Immediate cranial computed tomography scan showed small ischemic foci.

**Diagnosis:**

Initial diagnosis revealed acute cerebral infarction as the symptoms mimicked stroke at onset. Furthermore, his symptoms progressed and magnetic resonance scan after 6 days of onset appeared negative on diffusion weighted imaging. Other etiologies were also then considered. Japanese encephalitis virus immunoglobulin M in the serum supported positive diagnosis of JE.

**Interventions:**

The patient was given Ribavirin, and then his symptoms slowly improved.

**Outcomes:**

Brain MRI on day 29 after the onset revealed high-intensity lesions in bilateral thalamus on diffusion weighted imaging. During the follow-up (at about 2 months after the onset), the patient's consciousness was clear but could not walk. At about 6 months after the onset, he could walk with parkinsonian features.

**Conclusion:**

Diagnosis of JE that mimicked acute stroke at onset and with no fever can be challenging. Recognition of disease development, MRI and Japanese encephalitis virus immunoglobulinM findings are helpful in early definitive diagnosis.

## 1 Introduction

Japanese encephalitis (JE) is a serious viral infectious disease that is generally spread by Culex mosquitoes. Human beings are regarded as the ultimate incidental hosts, and neurological sequelae or even death might occur in seriously affected individuals. The initial manifestations might be diverse, ranging from behavioral abnormalities, seizures, disorientation, coma, to spastic paralysis with high grade fever.^[1]^ Hemiplegia is a rare manifestation seen during the initial stage of JE.^[2]^ We herein present a case report of an individual with left-sided limb weakness but with no fever. Initial brain computed tomography (CT) scan showed small ischemic foci, which mimicked ischemic stroke. However, cranial magnetic resonance (MR) scan was negative on diffusion weighted imaging (DWI) after 6 days of onset, and so the diagnosis of ischemic stroke was ruled out. As the disease progressed, the patient developed other neurological sequelae and fever. Finally, Japanese encephalitis virus (JEV) immunoglobulin M (IgM) in the serum on day 8 and abnormal signals of bilateral thalamus by MRI on day 29 of onset highly supported the diagnosis of JE.

## 2 Case report

A 52-year-old man presented to our Emergency Department with acute onset of left-sided limb weakness for 2 hours. He did not have fever or cold before the onset he had a 5-year history of hypertension. Immediate cranial (CT) scan showed small ischemic foci. He was initially given aspirin and Clopidogrel without tissue plasminogen activator (tPA) thrombolysis.

He was immediately sent to our hospital in the West. Physical examination showed that he had clear consciousness. Neurological examination revealed that the left limb muscle strength was level 4 and meningeal irritation signs were negative. He was given treatment for acute ischemic stroke. But his symptoms gradually progressed to unclear speech and dysphagia after 24 hours. After 2 days, he was transferred to our hospital because of degeneration of left-sided weakness and altered level of consciousness for 5 hours. His vital signs were temperature of 38.0°C and blood pressure of 226/114 mm Hg. His consciousness was unclear and breath sounds indicated dry and moist rales. The pupil was isochoric and light reflex remained sensitive. The left upper limb showed no activity. His muscle tension, especially that of left limbs remained high.

**Figure 1:**
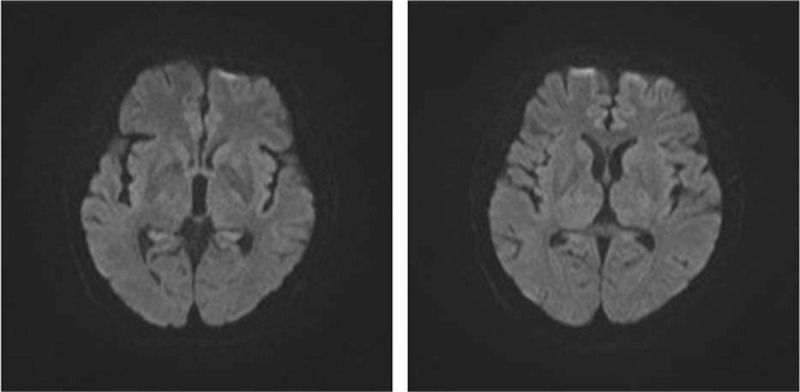
At 6 days after onset, there were no abnormal signals on diffusion weighted imaging.

Laboratory data at this time showed white blood cells of 14.47∗10^9^/L. Chest CT revealed inflammation in both lungs. He was treated for acute progressive ischemic stroke (brain stem) and pneumonia. However, his condition sharply deteriorated with drop in the oxygen level (SPO_2_) and he developed a stiff neck. Six days after the onset of JE, surprisingly, his MRI scan revealed no abnormal signals on DWI (Fig. 1). MR angiography revealed no remarkable occlusion or stenosis. The diagnosis was reconsidered as intracranial infection and the patient was given Ganciclovir.

**Figure 2:**
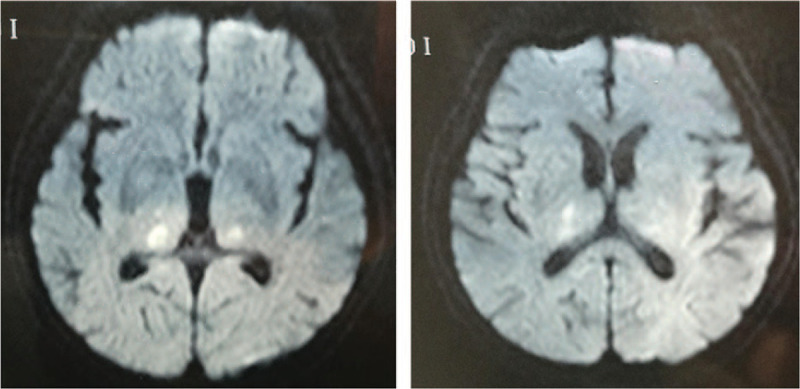
At 29 days after onset, diffusion weighted imaging images revealed high-intensity lesions in the bilateral thalamus.

The next day, lumbar puncture revealed that cerebrospinal fluid pressure was 165mmH_2_O, and the cell count was 93∗10^9^/L (98% mononuclear leukocytes and 2% polymorphonuclear leukocytes) with a protein level of 0.51mmol/L, and glucose level of 5.27mmol/L. The India-ink immunoreaction and autoimmune encephalitis antibodies were found to be negative. Analysis of herpes simplex virus DNA, Cytomegalovirus DNA, EB virus DNA, and tubercle bacillus DNA showed negative results. But JE IgM in the cerebrospinal fluid was not tested. However, JE related IgM in the serum was positive 2 days later. Electroencephalography (EEG) was abnormal on video EEG, which showed disappearance of the background rhythm and δ, θ rhythm diffuse distribution. He was diagnosed with JE finally. The patient was then transferred to other hospital and then given Ribavirin. DWI images still revealed no abnormal signals on day 19 after the onset. His symptoms were slowly getting better after treatment for 17 days in that hospital, and he was then discharged. He was sent to another hospital for rehabilitation. Brain MR imaging (MRI) on day 29 after the onset revealed high-intensity lesions in bilateral thalamus on diffusion weighted images (Fig. 2).

During follow-up (at about 2 months after onset), the patient had clear consciousness, but could not walk. At about 6 months after the onset, he could walk with parkinsonian features characterized by masking of face, paucity of blinking, and rigidity with tremors.

## 3 Discussion

JE is one of the most serious viral infectious diseases of the central nervous system in the world, especially in the Eastern and Southeastern Asia. Neurological sequelae or even death might occur in the affected individuals.^[3,4]^ The mortality associated with JE is estimated to be about 15,000 cases annually.^[5]^

The clinical manifestations of JE are non-specific, and other acute manifestations include behavioral abnormalities, seizures, disorientation, coma, and spastic paralysis that might lead to difficulty in diagnosis. Therefore, laboratory confirmation is necessary for diagnosing JE. The optimal method for laboratory confirmation is by testing JEV-specific IgM antibodies in the cerebrospinal fluid or serum.^[1]^ However, IgM titers are 100% detectable after 7 days of infection.^[6]^ Therefore, there might be a delay of 7 days at least before JE confirmation. JEV usually affects the diencephalon and the mesencephalon. Typical MRI results reveal abnormal signals in the thalamus (94%), basal ganglia (35.5%), midbrain (58%), cerebellum (25.8%), pons (19%), and cerebral cortex (19%).^[7]^ A very important imaging finding of JE is that the lesion usually first involves the posterior thalamus and then progresses to the whole thalamus and basal ganglion. Encephalitis with bilateral thalamic involvement is a highly suspicious sign of JE.^[8]^ However, in the present case, MR scan was negative after 6 days of onset.

In this case, it was difficult to distinguish JE from acute ischemic stroke initially. A diagnosis of cerebral infarction was made initially, which is a more common disease when the patient without fever had hemiplegia and hypertension; especially, cranial CT scan showed small ischemic foci within 24 hours. Now, there is a question: Is it wrong if the doctor administered the patient r-tPA thrombolysis when he presented with hemiplegia within 2 hours of onset? We do not think it wrong because alteplase is recommended for selected patients who might be treated within 3 hours of ischemic stroke symptom onset (Class I; LOE A).^[9]^ Furthermore, his symptoms have been progressed to unclear speech, dysphagia and unclear consciousness with fever. We misinterpreted these as symptoms of progressive stroke (brain stem) and aspiration pneumonia. The absence of diffusion-restriction lesion in initial MRI helped to exclude acute ischemic stroke as the reason of neurological symptom. With the suspicion of other etiologies, lumbar puncture was performed, which yielded abnormal results. Diagnosis of JE was then made because of the presence of JEV IgM in the serum on day 8 after the onset. The abnormal signals of bilateral thalamus on MRI on day 29 of onset highly supported the diagnosis of JE.

In conclusion, JE diagnosis that mimic acute stroke onset without fever can be challenging. Recognition of disease development, MRI and JEV IgM findings are helpful in early definitive diagnosis, treatment and improvement of the prognosis.

## Author contributions

**Conceptualization:** Jiali Zhao, Yifeng Du.

**Data curation:** Jiali Zhao, Fudi Chen, Lin Lu, Chunxia Li.

**Formal analysis:** Jiali Zhao, Fudi Chen, Lin Lu, Chunxia Li, Yifeng Du.

**Investigation:** Jiali Zhao.

**Methodology:** Jiali Zhao.

**Project administration:** Yifeng Du.

**Writing – original draft:** Jiali Zhao.

**Writing – review & editing:** Jiali Zhao, Fudi Chen, Lin Lu, Chunxia Li, Yifeng Du.
